# Enhancement of Mechanical Properties of Flax-Epoxy Composite with Carbon Fibre Hybridisation for Lightweight Applications

**DOI:** 10.3390/ma13010109

**Published:** 2019-12-25

**Authors:** Hom Nath Dhakal, Mohini Sain

**Affiliations:** 1 Advanced Materials and Manufacturing (AMM) Research Group, School of Mechanical and Design Engineering, University of Portsmouth, Portsmouth PO1 3DJ, UK; 2 Center for Biocomposites and Biomaterials Processing, Department of Mechanical and Industrial Engineering, University of Toronto, Toronto, ON M5S 3B3, Canada; m.sain@utoronto.ca

**Keywords:** Hybrid composites, carbon fibre, tensile properties, delamination, fibre-matrix interface

## Abstract

The effect of unidirectional (UD) carbon fibre hybridisation on the tensile properties of flax fibre epoxy composite was investigated. Composites containing different fibre ply orientations were fabricated using vacuum infusion with a symmetrical ply structure of 0/+45/−45/90/90/−45/+45/0. Tensile tests were performed to characterise the tensile performance of plain flax/epoxy, carbon/flax/epoxy, and plain carbon/epoxy composite laminates. The experimental results showed that the carbon/flax fibre hybrid system exhibited significantly improved tensile properties over plain flax fibre composites, increasing the tensile strength from 68.12 MPa for plain flax/epoxy composite to 517.66 MPa (670% increase) and tensile modulus from 4.67 GPa for flax/epoxy to 18.91 GPa (305% increase) for carbon/flax hybrid composite. The failure mechanism was characterised by examining the fractured surfaces of tensile tested specimens using environmental scanning electron microscopy (E-SEM). It was evidenced that interactions between hybrid ply interfaces and strain to failure of the reinforcing fibres were the critical factors for governing tensile properties and failure modes of hybrid composites.

## 1. Introduction

Composite materials reinforced with carbon and glass fibres have grown in popularity over the last few decades due to their advantageous properties and decreased cost compared to their metal counterparts. However, the ever-worsening condition of our planet because of unprecedented climate change is becoming an increasingly pressing issue, and as a result, new environmental legislations have redirected research into more sustainable, cost effective, and environmentally friendly lightweight composite materials [1,2,3]. 

There are different types of natural fibres that have been used in composite reinforcements. The commonly used fibres include hemp, flax, jute, kenaf, and sisal. Natural fibre-reinforced polymer composites are emerging as alternative materials in many non-structural engineering applications because of their higher specific properties (strength and modulus) and good ecological attributes compared to their conventional counterparts, such as carbon and glass fibre-reinforced composites. However, their susceptibility to moisture absorption, variation in their properties, poor mechanical properties due to weak fibre-matrix interface, and low fire resistance have been a hindrance for their use in high-performance structural applications [4,5,6,7].

Many attempts have been made over the years to improve the fibre matrix interface and water repellence behaviour of natural fibre composites by using various processes including chemical and physical fibre treatment methods, such as [8,9,10]. The findings from these works suggested that appropriate chemical and physical treatments such as alkalisation by using sodium hydroxide (NaOH), acetylation, cyanoethylation, silane coupling agent, and heating can reduce water absorption behaviour and improve mechanical and thermal properties of natural fibre composites through the enhancement of interfacial properties [11,12,13]. 

In recent years, there have been a significant number of research works undertaken dealing with the use of hybrid techniques in order to improve the properties of natural fibre-reinforced composites [14,15]. Hybridisation between natural and synthetic fibres is a technique in which the benefits of each material can be combined to achieve a composite that can demonstrate both higher mechanical performance and improved environmental features [16]. The work carried out by Fiore et al. [17] using basalt fibre as external hybridising material in flax/epoxy composites reported that because of a hybrid effect there was a significant improvement in flexural and impact properties. Recent works on hybrid effect by Almansour et al. [18] and Li and Sain [19] reported that natural fibre hybridised with glass and basalt fibre composites provided improved fracture toughness as well as stiffness and tensile strength. There were several reported works investigating the effects of glass and basalt fibres hybridisation on the performance of natural fibre-reinforced composites [20,21,22,23,24,25]. These reports on hybrid composites revealed that both basalt and glass as hybrid materials played a synergic role in improving various mechanical properties (tensile, flexural, fatigue, and impact) and thermal properties (glass transition temperature, improved degradation behaviours) [26,27]. 

Carbon fibres have become an important reinforcing material for many lightweight composite applications such as aerospace, automotive, and sports equipment due to their low density, high tensile strength and modulus, and lower susceptibility to corrosion (Table 1) [28,29]. However, carbon fibres are created from unsustainable fossil-based materials through energy intensive processes. As a result, products manufactured from carbon fibre-reinforced composites have a large carbon footprint [30]. Through life cycle assessment techniques, it was shown that 1 kg of carbon fibre composite consumes up to 300 MJ of energy for production [31]. In addition to this, limited recyclability and non-biodegradability of carbon fibre have become a growing concern when disposing of waste end of life products. Studies carried out by Das et al. [32] highlighted that wood and biochar biocomposites exhibited highest mechanical properties (tensile and flexural) and improved fire resistant behaviour when compared to other waste biomasses. Additionally, hybridisation was used to improve mechanical properties as well as limiting oxygen index of waste-based biochar/wood hybrid composites [33].

Due to their superior properties, carbon fibres are often hybridised with natural fibre composites in order to create composites with balanced properties. The work carried out by Dhakal et al. [34] on hybridisation of carbon fibre into flax fibre epoxy composites reported that more ductile behaviour could be realised through hybridisation. Moreover, their work revealed that water repellence behaviour and thermal properties of flax fibre composite was significantly improved through hybridisation with carbon fibre. There is clear evidence from the literature that the hybrid approach can offer a synergistic effect and provide the best properties of each of the constituent components in the resultant composites. However, for the hybrid systems to be effective, the compatibility of hybridising constituents, how they fail and an understanding of their structural performances relating to hybridisation are important. In this regard, there are not many reported works highlighting the failure modes and damage mechanisms of hybrid composites. In particular, there are insufficient reported works investigating carbon fibre hybridisation with natural flax fibres, as well as analysing their synergetic effects on light-weight critical applications. 

Flax (*Linum usitatissumum*) is a widely used natural fibre. The average chemical composition of flax fibre is cellulose (71%), hemicellulose (19.6%), while other constituents are pectin (2.2%), lignin (2.2%), and wax (1.5%) [10,35]. It is an attractive reinforcement material due to its several attractive properties such as specific tensile strength and modulus compared to conventional glass fibres. However, for natural fibre-reinforced composites to be used in light weighting semi-structural and structural applications such as automotive, marine, and aerospace, their mechanical properties such as tensile strength and modulus need to be improved so that the design specifications assigned by the Original Equipment Manufacturers (OEMs) are met and their damage mechanisms are understood. 

A recent comprehensive review on the lightweight application of composites carried out by Pervaiz et al. [1] argued that greenhouse gases (GHGs) generated by automotive vehicles counts for more than a quarter of all GHGs generated. Despite several drawbacks of carbon fibres in terms of their poor environmental performances, they strongly suggested that the lightweight and excellent mechanical properties of carbon fibre can help in reducing vehicle weight significantly, and hence help in reducing the overall CO_2_ emission. Taking this scenario into consideration and as a motivating factor, this study focuses on the experimental investigation into the effects of carbon fibre hybridisation on the tensile properties of flax fibre-reinforced epoxy composites. In order to understand the critical factors for improving mechanical performance of carbon/flax hybrid composites, the present work further assesses the damage mechanisms on the fractured surfaces of plain flax and plain carbon composites in comparison with the damage mechanisms of carbon/flax hybrid systems by using environmental scanning electron microscopy (E-SEM). 

## 2. Experimental Procedures

### 2.1. Materials 

The two reinforcing materials used for this investigation were epoxy-based prepregs ‘HexPly M56′ unidirectional carbon fibre (supplied by Gurit, UK) and ‘SDH VTC401LV’ unidirectional flax fibre (supplied by SDH Composites, Sinfin Derby, Derbyshire, UK). Prepregs are especially formulated matrix systems reinforced with reinforcing fibres that are ready to use for laminate manufacturing. Both prepreg materials were stored in a freezer until 24 h prior to lay up when they were removed and de-frosted. The unidirectional carbon tape epoxy based prepreg, with a fabric weight of 280 g/m^2^, had a fibre density of 1.78 g/cm^3^. The flax fibre prepreg unidirectional mats, with a fabric weight of 350 g/m^2^, as well as the flax fibres, had a density of 1.5 g/cm^3^ with a resulting fibre volume of the prepreg equal to 50%.

Physical and mechanical properties of flax and carbon fibre are presented in Table 1.

### 2.2. Fibre Orientation and Contents

The three types of composite laminates comprised of flax alone, carbon alone, and a combination of carbon and flax were fabricated. The laminate configuration chosen was symmetrical and contained an equal number of plies (8) as 0/+45/−45/90/90/−45/+45/0. The details of laminate information are presented in Table 2. 

### 2.3. Composite Laminates Fabrication

#### 2.3.1. Hand Lay-up in Combination with Vacuum Bagging 

**Figure 1:**
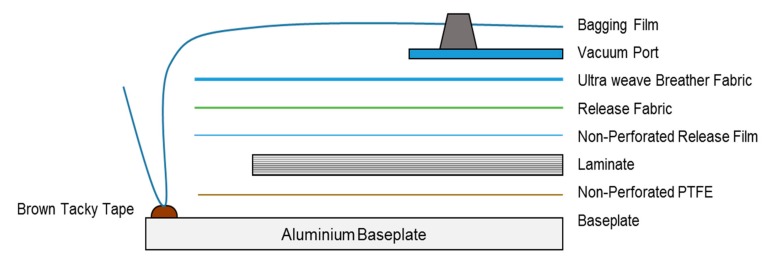
Schematic diagram of vacuum bagging.

Reinforcing plies were laid and a vacuum bag was created using bagging film, breather fabric, red release film, and brown tack tape. A ply compass was positioned next to each baseplate before the hand lay operation. The first ply of each panel was laid up using an L-shaped reference edge, once the correct position was achieved the poly backer was removed. Any visible air bubbles present on each ply were removed by hand. The same procedure was repeated for all remaining plies until the panel was completed. Vacuum consolidation was carried out after every 2 plies using the vacuum bag, a vacuum of 0.088 MPa was sustained over a period of 5 min. After all plies had been laid, a final vacuum consolidation of 0.088 MPa for at least 1 h was applied. Once all plies had been laid and the final vacuum consolidation had been completed, the laminates were covered with non-perforated release film to protect from contaminates. A schematic diagram of vacuum bagging is illustrated in Figure 1. Importantly, the fibre weight ratios for plain flax-reinforced composite, plain carbon composite, and carbon-flax hybrid composites were 100:0; 0:100 and 50:50, respectively.

#### 2.3.2. Cure Cycle

After the vacuum bag had successfully passed the vacuum drop test, the panels were cured in the curing oven. A standard oven cycle was used for carbon only laminates; however, due to the matrix used in the flax fibre, standard oven cure cycles were not applicable and a bespoke programme was written. The two oven cure parameters used are detailed as follows: 

Carbon only Laminates:

1. Full vacuum applied–1.00 bar.
2. Ramped from 20 °C to 180 °C ± 5 °C at 1–2 °C/min.
3. Dwelled at 180 °C ± 5 °C for 60 min (tolerance −5 min, +10 min).
4. Cooled from 180 °C to 40 °C at 0.5–5 °C/min.

Flax only and Flax-carbon hybrid laminates:

1. Full vacuum applied–1.00 bar.
2. Ramped from 20 °C to 135 °C ± 5 °C at 1–2 °C/min.
3. Dwelled at 135 °C ± 5 °C for 60 min (tolerance −5 min, +10 min).
4. Cooled from 135 °C to 40 °C at 0.5–5 °C/min.

The two samples were processed under similar conditions. The only difference was their ramping and dwelling temperatures, which were from 20 °C to 180 °C ± 5 °C and 180 °C ± 5 °C, respectively for CFRP composite sample, and 20 °C to 135 °C ± 5 °C and 135 °C ± 5 °C for FFRP and its hybrid samples. These temperatures were effective to obtain expected full curing. These curing parameters were recommended in the materials data sheets supplied by their manufacturers.

### 2.4. Tensile Testing

Tensile test specimens were individually machined from the laminate slabs using water jet cutting. The tensile tests were performed using an Instron UTM Model 3367 (Norwood, MA, USA) at room temperature, at a crosshead speed of 5 mm/min and with a preload of 0.5 N. The specimen dimensions and crosshead speed were selected in accordance with the BS standard [36]. The length of the specimen was 150 mm and the gauge length was 60 mm. The applied force versus extension of the specimen was recorded until the sample fully failed. No tabs were utilised and the breaks occurred within the gauge length. The tensile strength and modulus of the composites were calculated using the standard method, and for each composite type, five tests were carried out and the average values are used for discussion.

### 2.5. Damage Characterisation

The fractured surfaces of the different composites were examined using environmental scanning electron microscopy (E-SEM, Quanta FEG250, The FEI Quanta™, USA) equipped with X-ray energy dispersive spectroscopy detector (EDS). This is an advanced technique, which is well suited to the hydrophilic nature of natural fibre-reinforced composites, and importantly, it allows wet samples to be imaged without conductive coating.

## 3. Results and Discussion

### 3.1. Tensile Strength and Modulus

**Figure 2:**
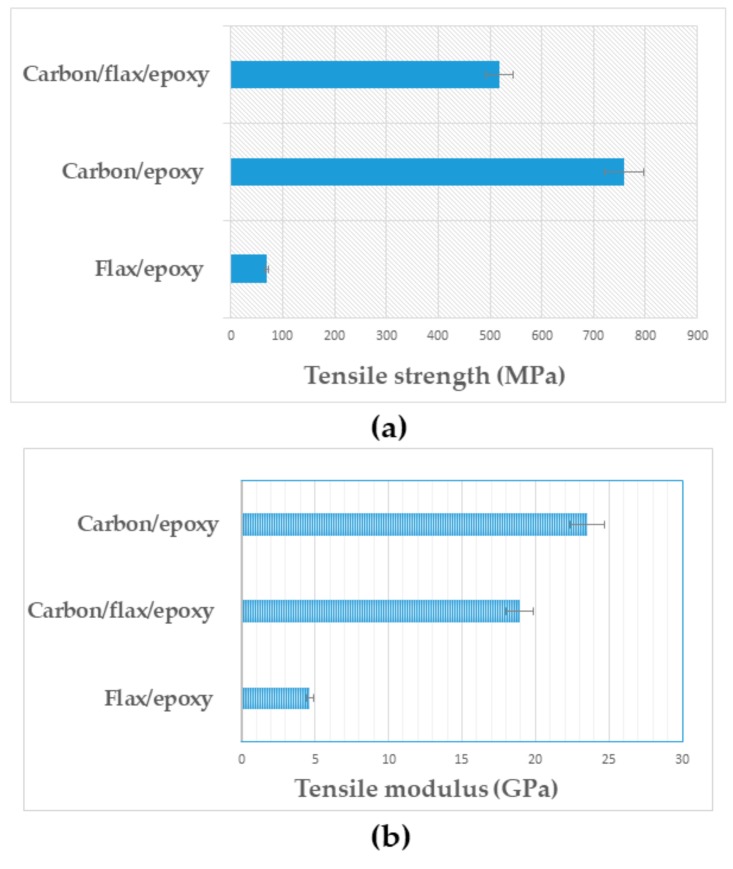
Comparative results of tensile strength (**a**) and modulus (**b**) for different composites.

The tensile properties including tensile strength, tensile modulus, and maximum extension of plain flax-reinforced composite, plain carbon composite, and carbon-flax hybrid composites at various weight ratios (i.e., 100:0; 0:100 and 50:50) were investigated. Among others, tensile strength and modulus are important properties of the material for design information, which mainly depend on the application and expected performance of the materials. The tensile test results obtained via calculation involving the load versus extension curves and specimen dimensions are summarised in Figure 2.

Figure 2a represents the comparative tensile strength for the composites studied. Plain carbon fibre composite displayed the highest tensile strength at approximately 759 MPa. The tensile strength recorded for plain flax and carbon flax hybridised composites was approximately 68 and 518 MPa, respectively.

Figure 2b depicts the results of tensile modulus for different composites. The modulus value for plain flax and plain carbon composites was recorded as approximately 4.67 and 18.91 GPa, respectively. The tensile modulus recorded for carbon fibre hybrid composite was approximately 23.52 GPa. With the hybridisation of carbon into flax composite, the tensile modulus of plain flax composite was increased from approximately 4.67 GPa to 23.52 GPa, an increase of approximately 400%. This significant increase in tensile properties was attributed to the layup of carbon fibre plies on the top and bottom layers of the hybrid laminates that helped to withstand the majority of the load applied on the laminates during tensile loading. The carbon-epoxy composite would be expected to provide highest modulus; however, it is noteworthy that the carbon flax hybrid system has even surpassed the stiffness value of plain carbon composites. Therefore, the hybrid system seems more beneficial for stiffness or modulus than the strength. It is reasonable to point out that the stiffness is related more to reinforcing effects, whereas the tensile strength is more related to the interfacial interaction between hybrid plies. Generally, natural fibres such as flax possess defects and dislocations in their structural morphology. These further contributed to the lowest tensile strength of flax-epoxy composite. However, these shortcomings were improved by the hybridisation of carbon fibre with the highest tensile strength. Consequently, this resulted in the highest tensile modulus of the flax-epoxy-carbon hybrid composite. 

The tensile strength and modulus of flax/carbon hybrid composites with carbon fibres at the outer layers contributed to higher values than flax/epoxy without hybridisation. This is due to the high load-carrying capacity of carbon fibres. The enhancement in the tensile strength and modulus of the hybrid composites compared to the flax composite without hybridisation was due to the addition of high modulus of carbon fibres compared to the flax fibres.

**Figure 3:**
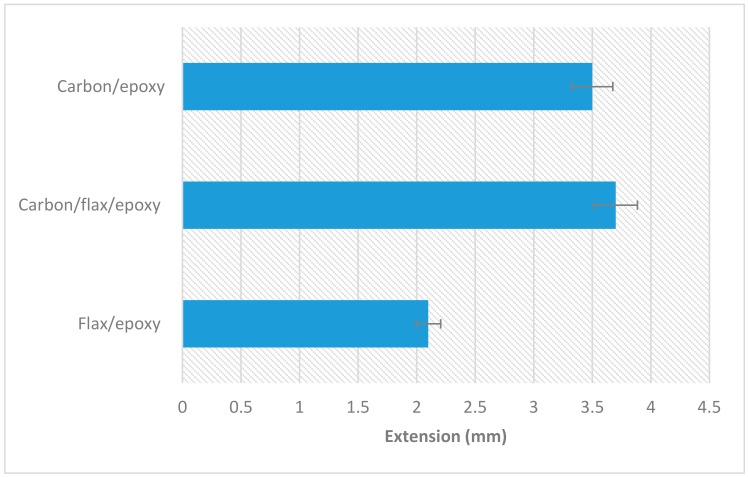
Comparative results for maximum extension at break for different composites.

The comparative results of extension at break for different composites are presented in Figure 3. The average extension at peak load recorded for plain flax and plain carbon composite samples was approximately 2.10 and 3.50 mm, respectively. With the hybridisation of carbon onto flax, the extension at peak load of flax/carbon hybrid composite achieved slightly higher than that of carbon/epoxy composite. The reasons for this improvement can be related to flax fibre having higher extension to break than carbon fibres. This can be further explained by the higher damping ratio of flax fibres compared to carbon fibres. The lowest value of extension for flax fibre/epoxy composite can be attributed to natural variability in the structure of flax fibre as well as presence of defects, kink-bands, and dislocations. Moreover, flax fibres often break prematurely, contributing to lower strength as well as lower extension of flax-epoxy composite. In addition, natural plant fibres (flax) have permeable walls, which allow epoxy resin to penetrate through and settle in the empty lumens during impregnation. This phenomenon makes flax epoxy absorb greater amount of epoxy resin than the carbon flax. 

### 3.2. Hybrid Effects on Failure Mode

**Figure 4:**
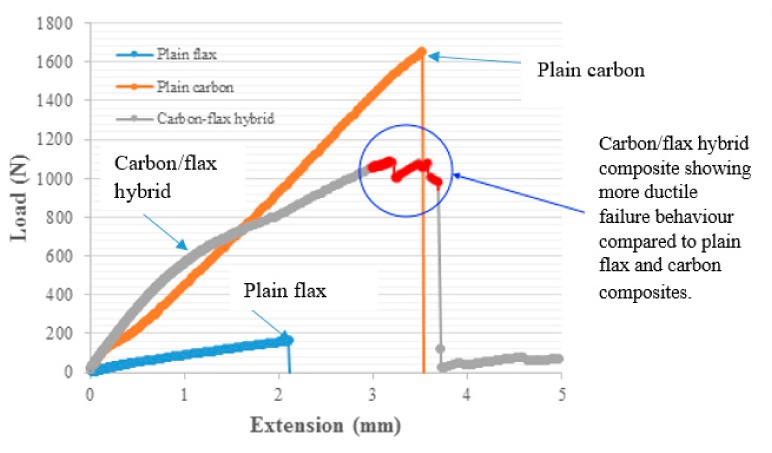
Load vs. extension curves for different composites.

**Figure 5:**
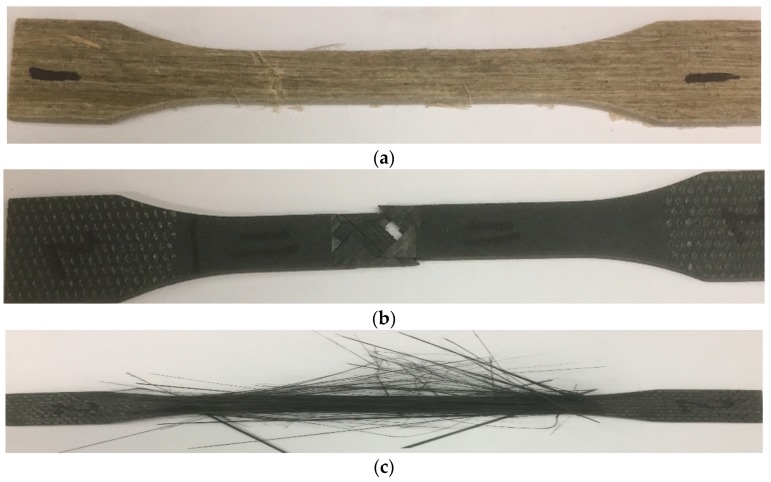
Representative tensile test specimens failed under tensile loading (**a**) plain UD flax/epoxy, (**b**) plain UD carbon/epoxy, and (**c**) UD carbon/flax hybrid composite. Abbreviations: UD, unidirectional.

**Figure 6:**
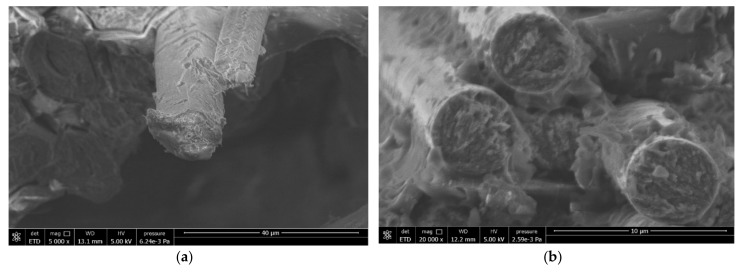
E-SEM images of fractured surface of (**a**) cross-sectional view of flax fibres and (**b**) carbon fibres.

Typical load-extension curves from the average of the five tests are illustrated in Figure 4. The load extension curve for the carbon fibre sample was linear up to its maximum breaking load and then there was a sudden drop. There was no any indication of damage initiation or damage load visible for the plain carbon epoxy composite. Representative fractured composite specimens after the tensile testing are shown in Figure 5. It is evident from Figure 5b that the plain carbon fibre composite failed in brittle fashion. Figure 6 depicts the comparative cross-sectional areas of fractured surfaces of plain flax composite and plain carbon composite. This brittle behaviour of carbon fibre was further evidenced by the E-SEM images of a fractured cross-sectional area. As evidently observed in Figure 6b, the fractured surface of the carbon composite showed a surface almost perpendicular to the load direction. Similar concern on the brittle failure behaviour of carbon composites was raised by Czel et al. [37]. They warned that this type of brittle failure behaviour of carbon fibre composites can limit their full usages in critical applications such as aerospace, automotive, and marine uses unless their failure behaviour, especially stress concentrations, are eliminated.

For carbon/flax hybrid samples, the load/extension curves show a non-linear behaviour with a relatively prolonged load extension curve. The non-linear behaviour for hybrid composited was attributed to high-strength carbon fibres reinforced on the outer layers of the hybrid composite. As carbon fibre possess higher strength and stiffness than flax fibre.

For the carbon/flax hybrid specimens, there was a portion of non-linear and a portion of prolonged curve before it finally broke (Figure 4). 

The load/extension curve for carbon/flax hybrid composite (Figure 4) can be approximately divided into two parts. The first part was related to damage initiation where there was a linear increase of load with steep curve against the extension and at this state there would be no visible damage, indicating elastic response of the samples. The second part was mainly associated with the damage propagation phase where the slope of the load extension curve decreases incrementally and again prolongs before it finally fails. As for the plain flax sample, the second part of the curve, which is damage propagation, seems a lot less than that of carbon/flax hybrid sample. With the hybridisation of carbon with flax, the load extension curve changed from linear to more prolonged non-linear, indicating more ductile failure compared to plain flax and plain carbon. This specific behaviour can be related to hybrid effects at the interphases level of flax-carbon-epoxy hybrid composites.

**Figure 7:**
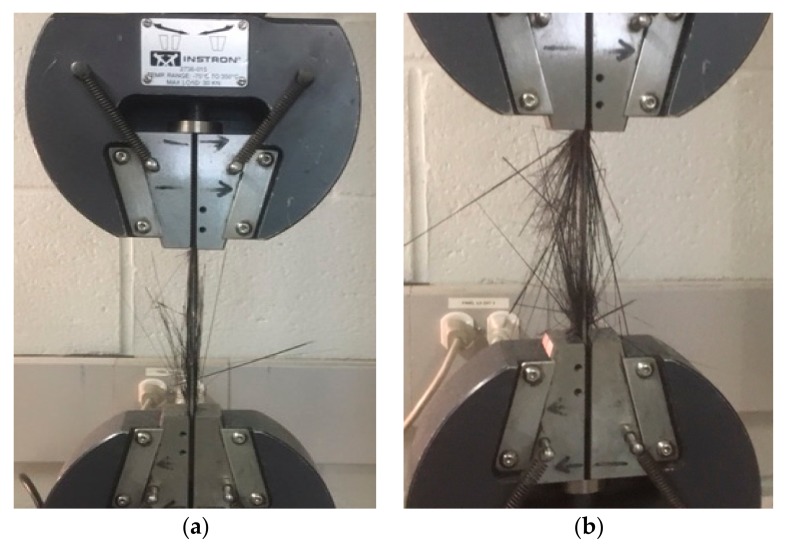
Fracture profile of tensile tested carbon flax hybrid composites (**a**): initiation of fibre rupture and (**b**) outer plies carbon fibre rupture.

Visual observation of broken tensile tested samples of carbon/flax hybrid composite shows the fragmented fibres under the tension loading. The fibres damage observed in Figure 7 suggests fibre rupture and fragmentation. The damage in tensile failure mode for carbon/flax hybrid epoxy specimen from the fractured cross-sectional area (Figure 6) and the visual observation of damaged fibre (Figure 7) suggest that it was due to fibre rupture. In this failure mode, matrix also contributes to the failure process. In this case, the matrix used was epoxy, which is a brittle polymer. Here, the combination of first matrix cracking and fibre rupture may have contributed to the fragmentation and rupture of carbon fibres.

### 3.3. Damage Characterisation

#### 3.3.1. Damage Characterisation of Flax/Epoxy Composite without Hybridization

**Figure 8:**
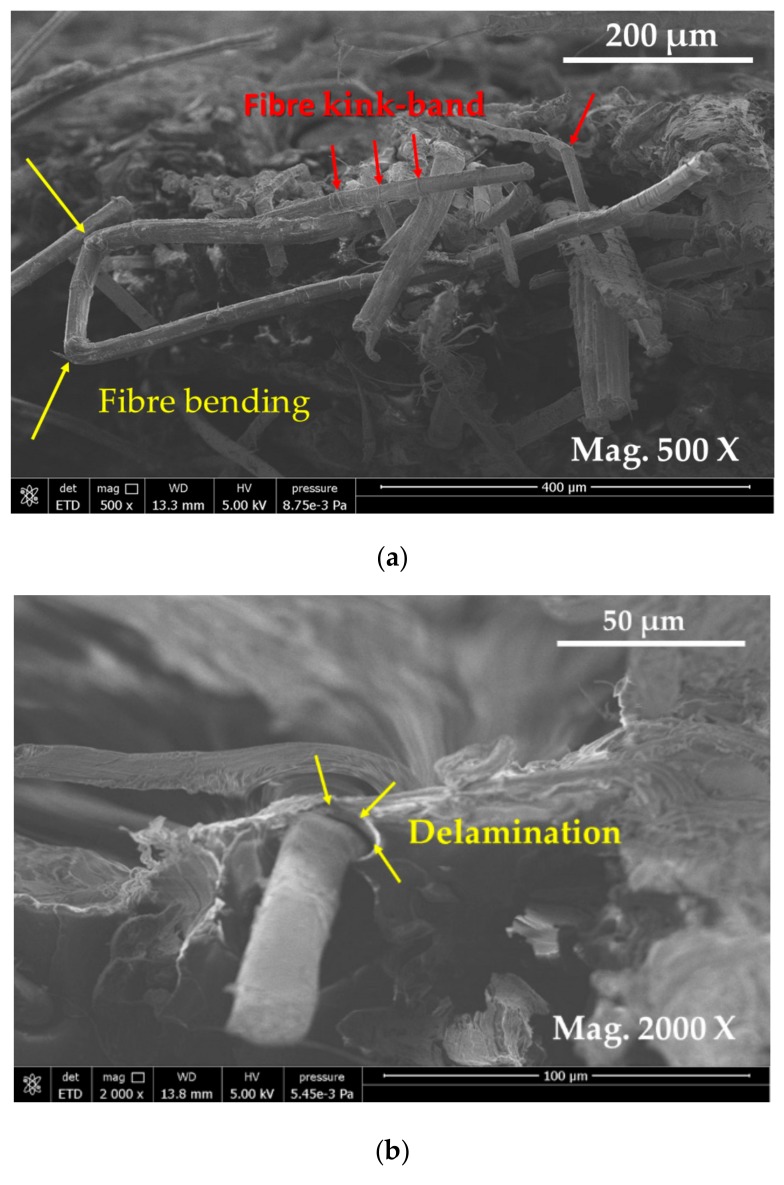

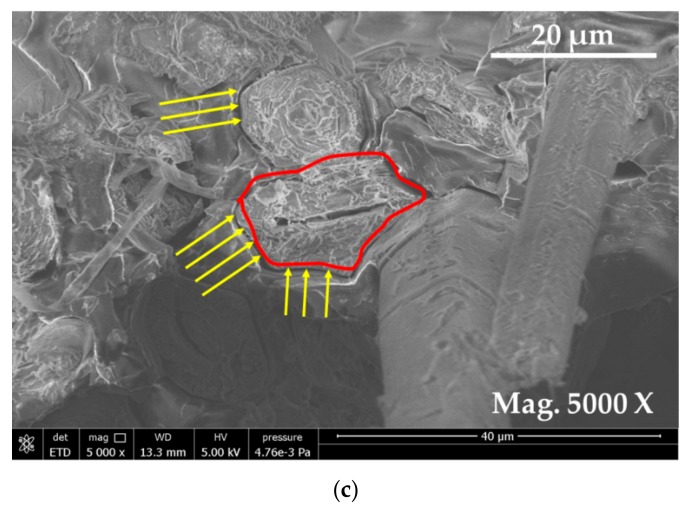
E-SEM micrographs of fractured surfaces of plain flax composites image showing (**a**) magnified view where flax fibres were observed bent in the nodes and flax fibre at the kink-band areas were observed in non-uniform diameter and with some impurities on the surfaces, nodes, and kink-bands observed clearly; (**b**) flax fibres observed delaminated from the matrix; and (**c**) magnified images of flax fibres showing lumen squeezed (laminated).

**Figure 9:**
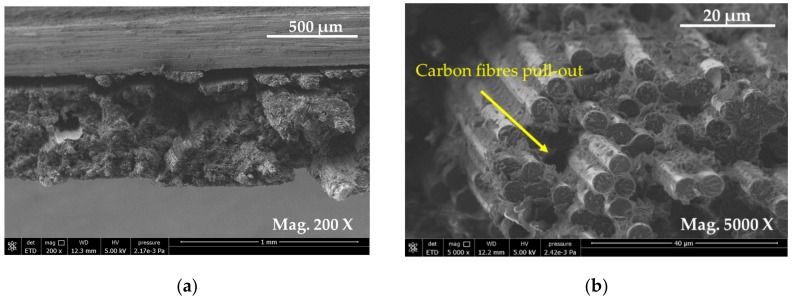
E-SEM micrographs of fractured surfaces of plain carbon fibre composites: (**a**) image showing carbon fibre aligned on the top at 0/90-degree orientation magnified view of carbon fibres observed aligned, and (**b**) magnified images of carbon fibres showing fracture in perpendicular direction of the applied load where fibre pull-out was observed.

E-SEM images of plain flax composites with different magnification are presented in Figure 8. The fractured surfaces of flax fibres reveal the presence of kink bands owing to fibre bending and buckling (Figure 8a). Due to the kink bands and nodes present, the flax fibres seem to have been damaged at these points. When the tensile load is applied, these points can act as stress concentration factors and the damage can initiate from these points. Fibre entanglements was also observed. This can be attributed to poor fibre arrangement during fabrication, which seemed to cause fibre buckling. In Figure 9a, longitudinal fibre split and cracks along the inner layers were also visible. In Figure 8a, some fibre bending on the kink band regions were visible. The work carried out by Notta-Cuvier et al. [38] suggest that flax fibre itself can be seen as a complex composite material which has several elementary fibres. This structure of flax fibre can influence the mechanical properties of resultant composites. Delamination was also observed (Figure 8b). Larger magnification (Figure 8c) shows an interesting feature where lumen of flax fibre was elongated, which may have occurred during the manufacture of flax laminates. These various parameters and variability affect the overall tensile properties of plain flax composites.

#### 3.3.2. Damage Characterization of Carbon/Epoxy Composite

Figure 9 presents E-SEM images of plain carbon composites samples at low and high magnifications. The E-SEM images (Figure 9a,b) showed a large gap between two adjacent plies of two different orientations (0/90). Delamination between these two adjacent plies were visible. In Figure 9a,b, some fibre pull out was also observed. Similarly, Figure 9b shows that the carbon fibres had broken in brittle fashion, as evidenced by their ends. At the higher magnification, (Figure 9b), the plain carbon composite showed brittle failure with evidence of damaged fibre ends perpendicular to the applied load. As expected, the damage behaviour in plain carbon composite seemed dominated by the fibre failure under the tension loading.

From Figure 9a,b, it is clear that the outer fibres have failed in the longitudinal direction. The fibre damage observed in Figure 7 showed fibre rupture and fragmentation. This is evidence that the damage of composites was dominated by the carbon fibre failure and rupture. In this, the damage seemed concentrated at carbon plies that were aligned at the outer surfaces, leading to a catastrophic failure (Figure 7 and Figure 8), as sudden load drop was clearly visible.

The delamination observed between adjacent plies was attributed to the inert surface of carbon fibres that can lead to problems such as poor interaction between matrix and inert carbon fibre surface. To minimise this problem, there were several surface modification methods being considered in the literature [39]. One of them is the chemical treatment of carbon fibre surface by improving their surface energy, which is suggested to be advantageous to promote the fibr-matrix interfacial interaction and wettability [40,41,42].

#### 3.3.3. Damage Characterisation of Carbon/Flax/Epoxy Hybrid Composite

**Figure 10:**
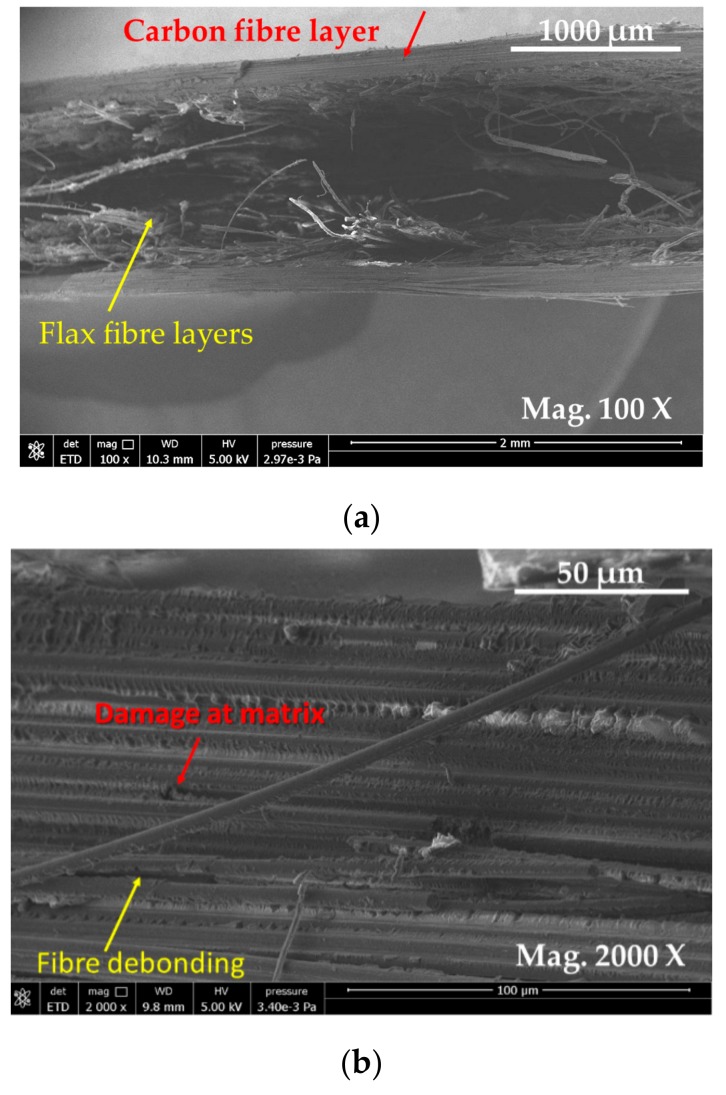

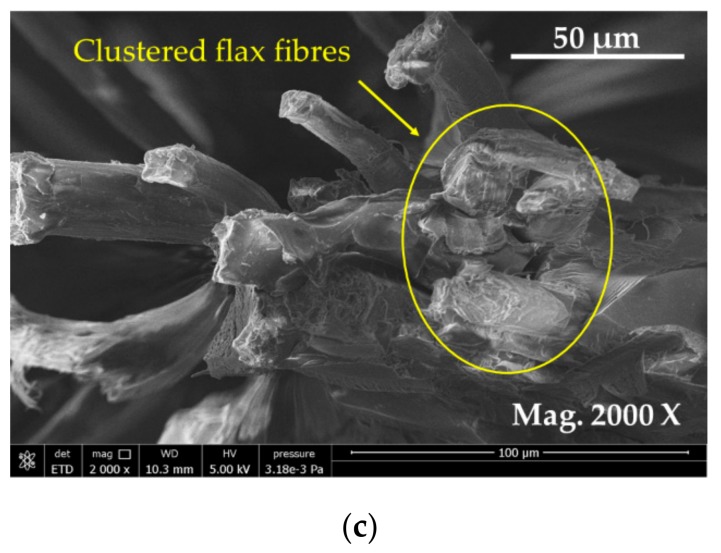
E-SEM micrographs of fractured surfaces of carbon flax hybrid composites: (**a**) image showing carbon fibre aligned on the top and bottom surfaces and flax being observed in the middle; (**b**) carbon fibre observed properly aligned, some defects on the surface of carbon fibre are visible and some delamination is also visible; and (**c**) flax fibres observed clustered in the middle.

E-SEM images of carbon-flax hybrid composite at different magnifications is presented in Figure 10. At the lower magnifications, Figure 10a, it was observed that carbon fibre plies are on the two outer layers, top and bottom of hybrid composite laminates. Two adjacent plies of carbon and flax did not seem well adhered where flax fibres are sandwiched between two plies of carbon fibres at the top and bottom of the laminate. Two damage modes were visible on carbon/flax hybrid composites: (1) damage at matrices, and at the interfaces between fibre and matrix; and the (2) delamination between the plies. It was also argued that fibre plies and matrix interface areas can experience large stress variation where micro-cracks can be initiated at the interfaces and promote accelerated crack propagation, reducing mechanical properties. Figure 10c shows flax fibres clustered in the middle of the sample.

The delamination of fibre plies at the interface was one of the issues that was encountered in the hybrid composites [43]. It was well established that the improvement in the tensile properties of composites was largely influenced by the effective load transfer at the fibre matrix interface [44]. There are several methods employed to improve fibre matrix interface of natural fibre-reinforced composites. By using these various treatments, the interaction between hydrophilic flax fibre and hydrophobic polymer can be significantly improved by removing natural and artificial impurities present on the surface of the flax fibres. The damage modes and the mechanisms of hybrid composites were also affected by the thermal residual strains arising from the mismatch of reinforcing constituents. 

## 4. Conclusions

This study has experimentally investigated the carbon fibre hybrid effects on the tensile properties and the damage mechanisms of UD flax/carbon composites. Following the results of tensile tests and the damage images obtained from E-SEM examination, the following conclusions can be drawn:

- There are several factors that influenced the tensile properties in hybrid composites; namely fibre plies orientation and the interactions between fibre plies, and the interfacial adhesion between the reinforcing fibres and the matrix.
- Overall, combining flax fibre with carbon fibre to form a hybrid composite improved tensile properties of flax/carbon composite significantly. More specifically, hybrid laminate significantly increased tensile modulus compared to plain carbon and plain flax laminates.
- Although carbon fibre-reinforced composite was proven to be superior to flax fibre-reinforced composite in terms of tensile strength and modulus, a 50/50 carbon/flax hybrid composite exhibited improved tensile properties whilst showing less brittle failure mode than plain carbon composites. This would also serve to attain light weighting agenda by using lower density materials where required mechanical properties of natural fibre composites can be enhanced by benefiting from outstanding properties of carbon fibres and at the same time, consumption of expensive and unsustainable petroleum-based resources can be minimised by using more sustainable and renewable flax fibres.
- The damage mechanisms for plain carbon composites were brittle where the carbon/flax hybrid composites showed less brittle and delamination was found to be the main failure mode. In the future, some surface modification could be considered for inert carbon fibre and flax fibre with an aim to improve overall hybrid interactions and reduce overall delamination in carbon/flax hybrid composites.
- The novelty of this work lies in the development of lightweight flax/carbon hybrid composites and assessment of their properties as well as correlate to damage mechanisms against fibre orientation and structures. The findings of this paper will help to achieve the aspirations of using sustainable, cost effective, and environmentally friendly lightweight composites as a viable alternative for automotive and marine sectors.

## Figures and Tables

**Table 1:** Comparative physical and mechanical properties of flax and carbon fibre [9,29].

Fibre Types	Density (g/cm^3^)	Tensile Strength at Break (MPa)	Tensile Modulus (GPa)	Elongation at Break (%)
Flax fibres	1.50	345–1100	27.6	2.7–3.2
Carbon fibres	1.78	3800	240	1.6
E-glass *	2.5	2000–3500	70	2.5

* For comparison purpose.

**Table 2:** Laminate information.

Panel Description.	Width (mm)	Length (mm)	No. of Plies	Cured Ply Thickness	Panel Thickness (mm)
Carbon Fibre	300	300	8	0.27	2.16
Flax Fibre	300	300	8	0.25	2.15
Hybridisation	300	300	8	C 0.27 F 0.25	2.10

## References

[1] Pervaiz M., Panthapulakkal S., Birat K.C., Sain M., Tjong J. (2016). Emerging trends in automotive lightweighting through novel composite materials. Mater. Sci. Appl..

[2] Holbery J., Houston D. (2006). Natural-fiber-reinforced polymer composites in automotive applications. JOM J. Min. Met. Mater. Soc..

[3] Dhakal H.N., Zhang Z.Y., Richardson M.O.W., Errajhi O.A.Z. (2007). The low velocity impact response of non-woven hemp reinforced unsaturated polyester composites. Compos. Struct..

[4] Assarar M., Zouari W., Sabhi H., Ayad R., Berthelot J.M. (2015). Evaluation of the damping of hybrid carbon–flax reinforced composites. Compos. Struct..

[5] Hughes M. (2012). Defects in natural fibers: Their origin, characteristics and implications for natural fibre reinforced composites. J. Mater Sci..

[6] Dhakal H.N., Zhang Z.Y., Richardson M.O.W. (2007). Effect of water absorption on the mechanical properties of hemp fibre reinforced unsaturated polyester composites. Compos. Sci. Technol..

[7] Shen Y., Zhong J., Cai S., Ma H., Qu Z., Guo Y., Li Y. (2019). Effect of temperature and water absorption on low-velocity impact damage of composites with multi-layer structured flax fiber. Materials.

[8] Rong M.S., Zhang M.Q., Liu Y., Yang G.C., Zeng H.M. (2001). The effect of fiber treatment on the mechanical properties of unidirectional sisal-reinforced epoxy composites. Compos. Sci. Technol..

[9] Islam M.S., Pickering K.L., Foreman N.J. (2010). Influence of alkali treatment on the interfacial and physico-mechanical properties of industrial hemp fibre reinforced polylactic acid composites. Compos. Part A Appl. Sci. Manuf..

[10] Faruk O., Bledzki A.K., Fink H.P., Sain M. (2012). Biocomposites reinforced with natural fibers: 2000–2010. Prog. Polym. Sci..

[11] Ibrahim N.A., Hadithon K.A. (2010). Effect of fiber treatment on mechanical properties of kenaf fiber-ecoflex composites. J. Reinf. Plast. Compos..

[12] Sain M., Suhara P., Law S., Bouilloux A. (2005). Interface modification and mechanical properties of natural fiber-polyolefin composite products. J. Reinf. Plast. Compos..

[13] Dhakal H.N., Zhang Z.Y., Bennett N. (2012). Influence of fibre treatment and glass fibre hybridisation on thermal degradation and surface energy characteristics of hemp/unsaturated polyester composites. Compos. Part B Eng..

[14] Essabir H., Bensalah M.O., Rodrigue D., Bouhfid R., Qaiss A. (2016). Structural, mechanical and thermal properties of biobased hybrid composites from waste coir residues: Fibers and shell particles. Mech. Mater..

[15] Fan W., Yuan L., D’Souza N., Xu B., Dang W., Xue L., Li J., Tonoy C., Sun R. (2018). Enhanced mechanical and radar absorbing properties of carbon glass fibre hybrid composites with unique 3D orthogonal structure. Polym. Test..

[16] Flynn J., Amiri A., Ulven C. (2016). Hybridized carbon and flax fiber composites for tailored performance. Mater. Des..

[17] Fiore V., Scalici T., Calabrese L., Valenza A., Proverbio E. (2016). Effect of external basalt layers on durability behaviour of flax reinforced composites. Compos. Part B Eng..

[18] Almansour F.A., Dhakal H.N., Zhang Z.Y. (2016). Investigation into Mode II interlaminar fracture toughness characteristics of flax/basalt reinforced vinyl ester hybrid composites. Compos. Sci. Technol..

[19] Li H., Sain M.M. (2003). High stiffness natural fiber-reinforced hybrid polypropylene composites. Polym. Plast. Technol. Eng..

[20] Almansour F.A., Dhakal H.N., Zhang Z.Y. (2017). Effect of water absorption on Mode I interlaminar fracture toughness of flax/basalt reinforced vinyl ester hybrid composites. Compos. Struct..

[21] Wei B., Cao H., Song S. (2010). Tensile behavior contrast of basalt and glass fibers after chemical treatment. Mater. Des..

[22] Sarasini F., Tirillò J., Valente M., Valente T., Cioffi S., Iannace S. (2013). Effect of basalt fiber hybridization on the impact behavior under low impact velocity of glass/basalt woven fabric/epoxy resin composites. Compos. Part A Appl. Sci. Manuf..

[23] Dhakal H.N., Sarasini F., Santulli C., Tirillò J., Zhang Z., Arumugam V. (2015). Effect of basalt fibre hybridisation on post-impact mechanical behaviour of hemp fibre reinforced composites. Compos. Part A Appl. Sci. Manuf..

[24] Kc B., Faruk O., Agnelli J.A.M., Leao A.L., Tjong J., Sain M. (2016). Sisal-glass fiber hybrid biocomposite: Optimization of injection molding parameters using Taguchi method for reducing shrinkage. Compos. Part A Appl. Sci. Manuf..

[25] Fiore V., Calabrese L., Bruzzaniti P., Valenza A. (2018). Bearing strength and failure behaviour of pinned hybrid glass-flax composite laminates. Polym. Test..

[26] Saidane E.H., Scida D., Assarar A., Ayad R. (2017). Damage mechanisms assessment of hybrid flax-glass fibre composites using acoustic emission. Compos. Struct..

[27] Petrucci R., Santulli C., Puglia D., Sarasini F., Torre L., Kenny J.M. (2013). Mechanical characterisation of hybrid composite laminates based on basalt fibres in combination with flax, hemp and glass fibres manufactured by vacuum infusion. Mater. Des..

[28] Pickering K.I., ArauanEfendy M.G., Le T.M. (2016). A review of recent developments in natural fibre composites and their mechanical performance. Compos. Part A Appl. Sci. Manuf..

[29] Faruk O., Bledzki A.K., Fink H.P., Sain M. (2014). Progress report on natural fiber reinforced composites. Macromol. Mater. Eng..

[30] Cheung H.Y., Ho M.P., Lau K.T., Cardona F., Hui D. (2009). Natural fibre-reinforced composites for bioengineering and environmental engineering applications. Compos Part B Eng..

[31] Suzuki T., Takahashi J. Prediction of energy intensity of carbon fiber reinforced plastics for mass-produced passenger cars. Proceedings of the Ninth Japan International SAMPE Symposium JISSE-9.

[32] Das O., Kim N.K., Hedenqvist M.S., Lin R.J.T., Sarmah A.K., Bhattacharyya D. (2018). An Attempt to Find a Suitable Biomass for Biochar-Based Polypropylene Biocomposites. Environ. Manag..

[33] Das O., Kim N.K., Sarmah A.K., Bhattacharyya D. (2017). Development of waste based biochar/wool hybrid biocomposites: Flammability characteristics and mechanical properties. J. Clean Prod..

[34] Dhakal H.N., Zhang Z.Y., Guthrie R., MacMullen J., Bennett N. (2013). Development of flax/carbon fibre hybrid composites for enhanced properties. Carbohydr. Polym..

[35] Bastra S.K., Lewin M., Pearce E.M. (1998). Other long vegetable fibres. Handbook of Fibre Science and Technology. Fibre Chemistry.

[36] (1998). Glass Fibre Reinforced Plastics-Tensile Test.

[37] Czel G., Jalavand M., Wisnom M.R. (2016). Hybrid specimens eliminating stress concentrations in tensile and compressive testing of unidirectional composites. Compos. Part A Appl. Sci. Manuf..

[38] Notta-Cuvier D., Lauro F., Bennani B., Nciri M. (2016). Impact of natural variability of flax fibres properties on mechanical behaviour of short flax fibre reinforced polypropylene. J. Mater. Sci..

[39] Anstey A., Vivekanandhan S., Rodriguez-Uribe A., Misra M., Mohanty A.K. (2016). Oxidative acid treatment and characterization of new biocarbon from sustainable *Miscanthus* biomass. Sci. Total Environ..

[40] Sharma S.P., Lakkad S.C. (2015). Impact behavior and fractographic study of carbon nanotubes grafted carbon fiber-reinforced epoxy matrix multi-scale hybrid composites. Compos. Part A Appl. Sci. Manuf..

[41] Jiang D.W., Xing L.X., Liu L., Yan X.R., Guo J., Zhang X., Zhang Q.B., Wu Z.J., Zhao F., Huang Y.D. (2014). Interfacially reinforced unsaturated polyester composites by chemically grafting different functional POSS onto carbon fibers. J. Mater. Chem. A.

[42] Zhang X.Q., Xu H.B., Fan X.Y. (2014). Grafting of amine-capped cross-linked polyphosphazenes onto carbon fiber surfaces: A novel coupling agent for fiber reinforced composites. RSC Adv..

[43] John M.J., Thomas S. (2008). Biofibres and biocomposites. Carbohydr. Polym..

[44] Qu Z., Pan X., Hu X., Guo Y., Shen Y. (2019). Evaluation of nano-mechanical behaviour on flax fiber metal laminates using atomic force microscope. Materials.

